# Isolated Rectal Neurofibroma: A Case Report and Literature Review

**DOI:** 10.7759/cureus.63323

**Published:** 2024-06-27

**Authors:** Zhexiang He, Shuja Khan, Arthur Slaton

**Affiliations:** 1 Internal Medicine, Conway Regional Health System, Conway, USA

**Keywords:** adult gastroenterology, gastrointestinal pathology, isolated neurofibroma, colonoscopy, neurofibroma

## Abstract

Neurofibromas are considered benign peripheral nerve sheath tumors containing Schwann cells, fibroblasts, and perineurial cells. They are commonly associated with familial disorders. Isolated colonic neurofibromas are very rare. In this report, we discuss a case of a patient who presented to the gastroenterology clinic with a week-long occurrence of abdominal pain and bleeding. She underwent a colonoscopy in which three sentinel polyps of benign appearance, ranging in size from 4 mm to 10 mm, were removed during the procedure. The pathology report indicated that the distal rectal polyp contained a submucosal neurofibroma with SOX10+, desmin-, CD117-, DOG1-, CD34+. While NF1-associated neurofibromas harbor the risk of malignant transformation into malignant peripheral nerve sheath tumors (MPNSTs), the malignancy potential for isolated colonic neurofibromas remains uncertain due to their rarity. The clinical significance of isolated colonic neurofibromas is yet to be defined; therefore, the optimal management strategy remains uncertain. Close monitoring is advocated to both exclude the possibility of neurofibromatosis and be vigilant about the risk of malignant transformation.

## Introduction

Neurofibromas were considered benign peripheral nerve sheath tumors containing Schwann cells, fibroblasts, and perineurial cells [1,2]. They commonly associate with familial disorders, which include neurofibromatosis type 1 (NF1), neurofibromatosis type 2 (NF2)-related schwannomatosis (formerly NF2), germline PDGFRA-mutant syndrome(formerly/neurofibromatosis 3b, INF/NF3b) intestinal neurofibromatosis, and multiple neuroendocrine neoplasia IIb [2,3]. NF1 and NF2 were both considered as autosomal dominant genetic syndromes. The incidence of NF1 is approximately 1:2600 to 1:3000 individuals, while the incidence of NF2 is approximately 1:25,000 individuals [4].

NF1 and NF2 genes are tumor suppressor genes that are located at different chromosomes. Mutations in the NF1 gene, located on chromosome 17 (17q11.2), are commonly linked to cardiovascular, musculoskeletal, and gastrointestinal symptoms. On the other hand, mutations in the NF2 gene, located on chromosome 22 (22q11-13.1), are commonly associated with central nervous system symptoms [5]. Up to 25% of patients with NF1 report gastrointestinal involvement, and patients with PDGFRA-mutant syndrome also report gastrointestinal involvement with multiple gastric gastrointestinal stromal tumors (GIST) and inflammatory fibroid polyps [3,5,6]. However, isolated colonic neurofibromas are very rare, and only a few cases have been reported in the literature during the last several decades (Table 1). Further clinical follow-up may be indicated for those patient populations as their clinical finding could be associated with those familiar disorders in the future.

In this report, we present a rare case of isolated colonic neurofibroma.

## Case presentation

The patient is a 55-year-old female with a medical history of coronary artery disease (CAD) with a stent in place and on clopidogrel therapy. She was referred to the gastroenterology clinic by her primary care doctor due to abdominal pain associated with bleeding that occurred for a week. The patient described experiencing left lower quadrant abdominal pain with severe cramping and constipation. During this period, she had a bowel movement with blood and pus, which alleviated her pain. The symptoms improved, and the patient denied experiencing any other issues such as fever, chills, weight change, etc. The episode occurred two months before she visited the clinic. She mentioned that her symptoms had resolved, and she was having regular daily bowel movements.

**Figure 1:**
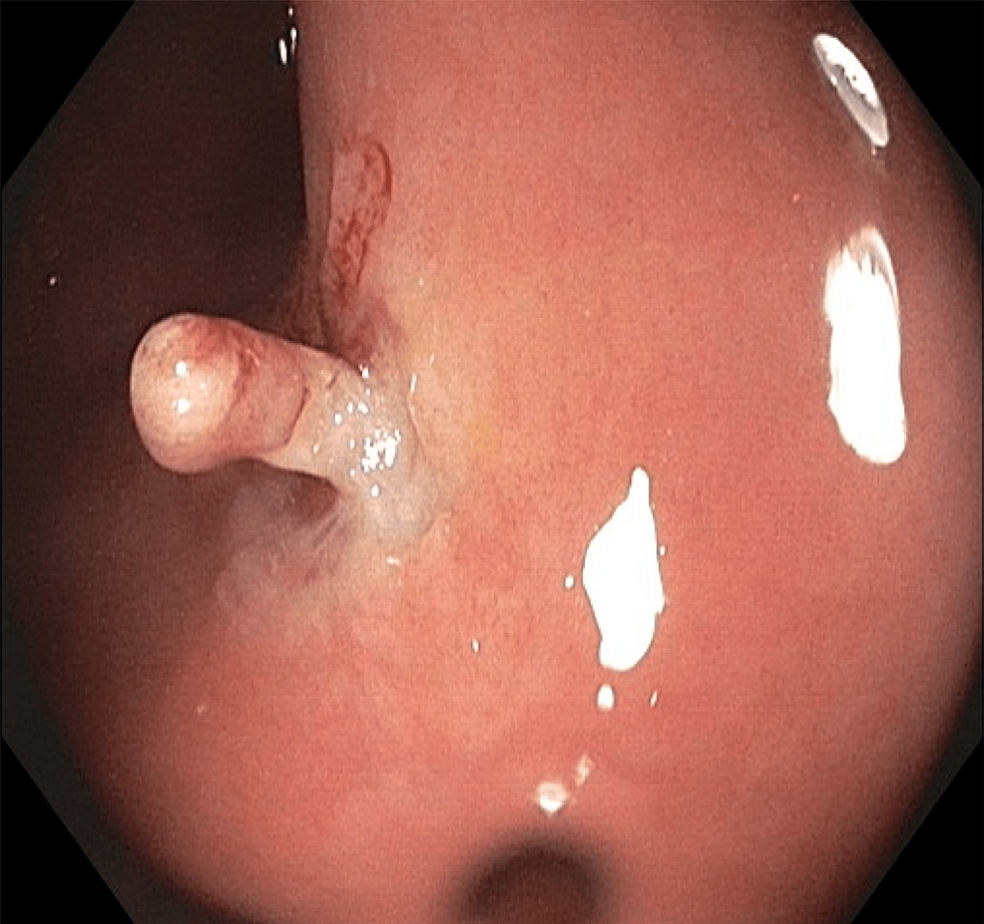
Patient colonoscopy shows a distal rectal polyp, which was identified as isolated rectal neurofibroma by pathology Source: Gastroenterology Department, Conway Regional Health System

**Figure 2:**
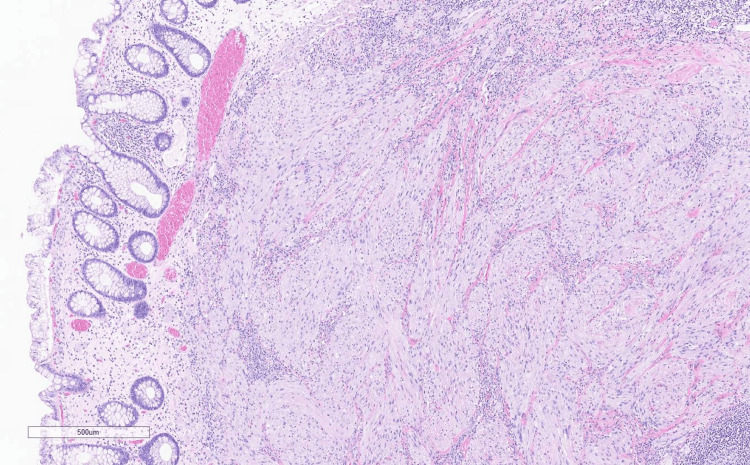
H&E shows spindled neurofibroma with characteristic histomorphology features of neurofibroma (non-plexiform).

**Figure 3:**
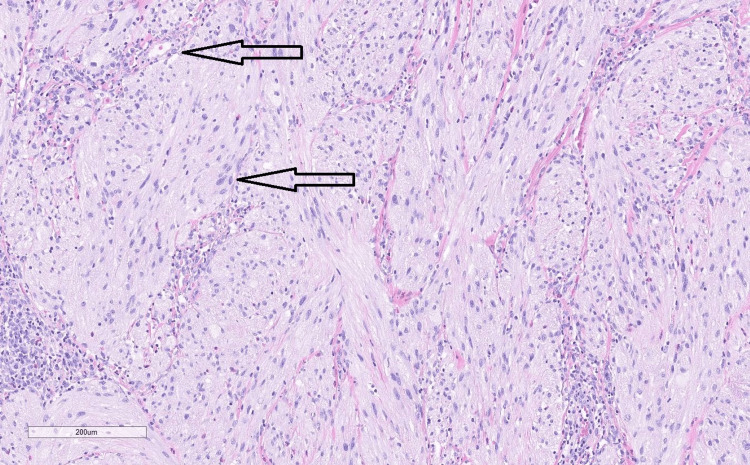
Polyp biopsy shows mast cells surrounding the tumor cells. H&E stain, 10X.

**Figure 4:**
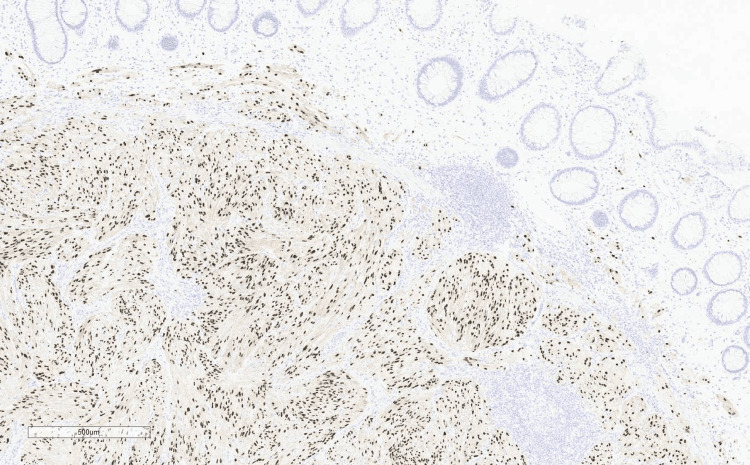
Patient polyp biopsy shows positive SOX-10 immunostain marker, used for identifying neural tumors

**Figure 5:**
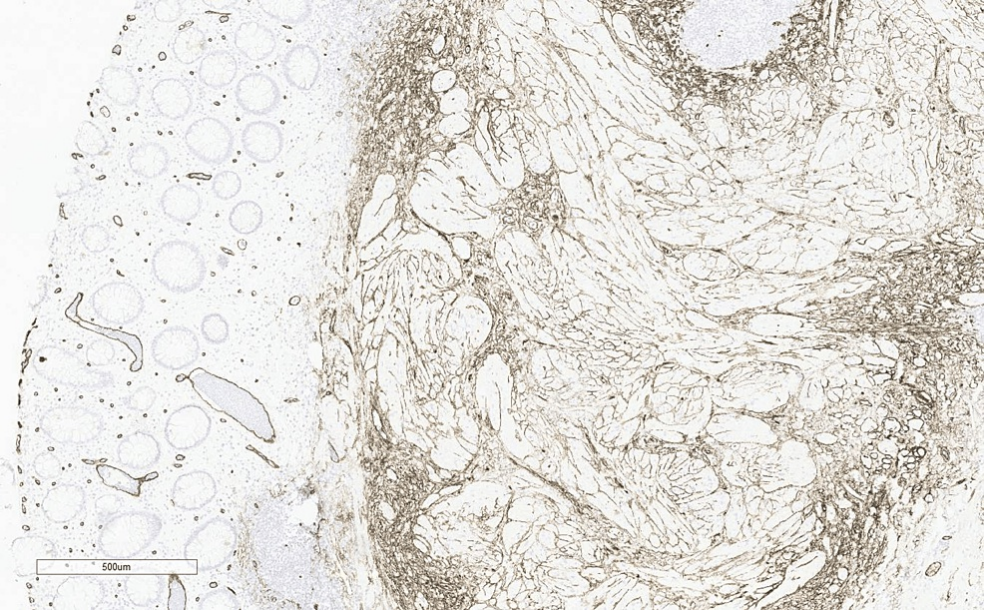
CD34 stain reveals a more loosely distributed staining pattern

**Figure 6:**
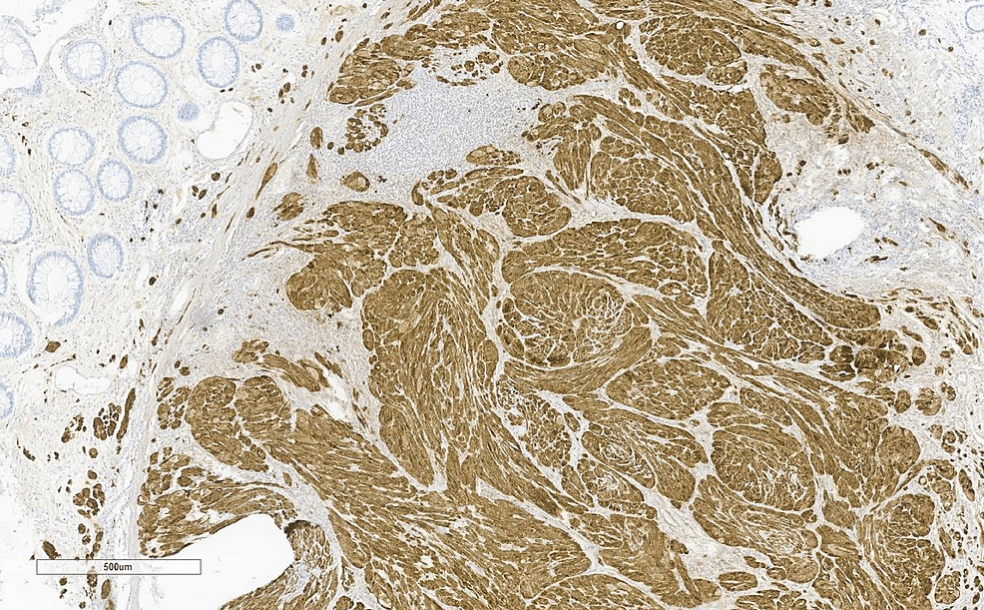
Polyp exhibits positive staining for S100

The patient had never undergone a colonoscopy in the past. After discussing the matter, she agreed to have a colonoscopy with the clinic. During the colonoscopy, multiple nonbleeding diverticula were found in the sigmoid colon, along with grade/stage II internal hemorrhoids. A single sessile 4 mm polyp with a benign appearance was detected in the mid-sigmoid colon. The polyp was completely removed through a single-piece polypectomy using a cold snare. In the distal rectum, three sentinel polyps of benign appearance, ranging in size from 4 mm to 10 mm, were discovered. Figure 1 shows a distal rectal polyp under the endoscope, the morphology of which was different compared to usual colonic polyps. These polyps were also completely removed through a single-piece polypectomy using a hot snare with an endoclip applied to establish hemostasis. The polyps were then sent for pathology which revealed that the polyps in the distal rectum were neurofibromas. Figures 2-6 show the pathological findings of the distal rectal polyp. 

During a follow-up visit, the patient denied any history of neurofibromatosis and stated that no neurofibromas had been removed from her extremities or skin. She was encouraged to discuss these findings with her primary care physician. We have discussed with the patient patient for a dermatology referral, and possible subsequent genetic counseling and optometry appointments if needed. A follow-up colonoscopy is scheduled at a one-year interval.

## Discussion

Neurofibromas represent benign tumors emerging from peripheral nerve sheath components, comprising Schwann cells, fibroblasts, and perineurial cells [27-31]. While multiple neurofibromas are commonly associated with hereditary syndromes like NF1 and NF2, isolated neurofibromas exist but are not well-described in the literature. Table 1 gives a summary of isolated colonic neurofibromas reported in the literature. This case discussion centers on these rare isolated neurofibromas.

**Table 1:** Isolated colonic neurofibromas with pathology reports SMA: smooth muscle actin; EMA: epithelial membrane antigen

Reference	Age	Sex	Presentation	Location	Pathology
Keith, et al. (1937) [7]	50	Female	Pain	Rectum	Benign spindle cell lesion
Woolf, et al. (1938) [8]	70	Male	Asymptomatic	Rectum	Spindle cell lesion with rare mitoses
Butler, et al. (1959) [9]	45	Female	Pain, bleeding, tenesmus	Rectum	Features of plexiform neurofibroma
Geboes, et al. (1978) [10]	N/A	N/A	NA	Rectum	Diagnosis confirmed by electron microscopy
Abramson, et al. (1997) [11]	53	Male	Rectal bleeding	Transverse colon	Neurofibroma S100 +
Bononi, et al. (2000) [12]	68	Female	Rectal bleeding, tenesmus	Diffuse involvement	Neurofibroma S100 + SMA -
Panteris, et al. (2005) [13]	65	Female	Bloody diarrhea Abdominal pain	Descending colon	Neurofibroma S100+, Vimentin+, Desmin –, SMA –, c-kit –, CD34 –
Carter, et al. (2008) [14]	52	Female	Non-bloody diarrhea, Pain	Rectum, transverse colon	Neurofibroma S100 +
Hindy, et al. (2012) [15]	59	Male	Screening colonoscopy	Transverse Colon	Neurofibroma S100 +
Chelimilla, et al. (2013) [16]	70	Female	Asymptomatic, Screening colonoscopy	Ascending colon	Neurofibroma S100 +, CD117 – SMA –, Desmin –
Bilal, et al. (2016) [17]	52	Male	Pain	Proximal descending colon	Neurofibroma S100 +
Ahn, et al. (2016) [18]	26	Female	Screening colonoscopy	Sigmoid colon	Neurofibroma S100 +, CD34+,c-Kit (CD117) –, DOG 1 –, SMA –
Kassi, et al. (2016) [19]	37	Female	Painful abdominal mass	Transverse colon	Neurofibroma
Parmar, et al. (2016) [20]	13	Female	Rectal bleeding	Rectum	NeurofibromaS100 +, c-Kit (CD117) –, desmin –
Adioui, et al. (2018) [21]	29	Fenale	Pain in left iliac fossa, Fatigue	Sigmoid colon	NeurofibromaS100 +, CD34 –, C- Kit (CD117) –, Desmin –
Miao, et al. (2018) [22]	24	Female	Pain, Mass in stool	Ileocecal valve	Neurofibroma S100 +, CD117 –, CD34 –, Actin –, desmin –, DOG 1 –, Low Ki 67
Olakayode, et al. (2018) [23]	12	Male	Painless left hypochondrial abdominal mass	Splenic flexure	Neurofibroma
Imagami, et al. (2020) [24]	81	Female	Positive fecal occult blood	Hepatic flexure	Neurofibroma S100 +, CD34 –, c-kit (CD117) –, desmin –
Ghoneim, et al. (2020) [25]	51	Male	Screening colonoscopy	15 cm from anal verge	Neurofibroma S100 +, Low Ki 67 index
Sun, et al. (2020) [26]	33	Female	Screening colonoscopy	Ascending colon	Neurofibroma S100 +
Tinguria, et al. (2022) [27]	76	Male	Anemia Symptoms of gastroesophageal reflux disease (GERD)	Sigmoid Colon	Neurofibroma Vimentin +, S100 +, EMA +, SOX 10 +, CD34 + (Patchy), Neurofilament + (in axonal elements), desmin –, CD117 –, Pan-cytokeratin (AE1/AE3) –, HMB 45 –, DOG 1 –
He et al. (2023) Current study	55	Female	Abdominal pain with bleeding	Distal Rectal Colon	spindled neurofibroma SOX10 +, desmin –, CD117 –, DOG1 –, CD34 ­+, S100+

NF1 and NF2 represent distinct clinical entities. NF1, often called von Recklinghausen's disease, encompasses features including six or more Café-au-lait spots that are larger than 0.5 cm in diameter before puberty or >1.5 cm post-puberty, two or more neurofibromas of any kind, or a single plexiform neurofibroma; axillary and inguinal freckling (Crowe's sign), Lisch nodules that are benign growths on the iris formed from melanocytes and do not impair eyesight, a distinctive osseous lesion such as sphenoid dysplasia, anterolateral bowing of the tibia, or pseudarthrosis of a long bone, optic Glioma, and a family history of NF1. An individual fulfilling at least two diagnostic criteria is considered NF1 positive [32-37]. This syndrome develops due to mutations in the NF1 gene on chromosome 17q11.2, which produces the neurofibromin protein. This protein functions as a tumor suppressor, ensuring cell proliferation remains in check [27,37]. Without functional neurofibromin, unchecked proliferation can lead to tumor formation. In contrast, NF2 mostly impacts the central nervous system [34].

Notably, evidence proved that some of the NF1 cases arise from sporadic mutations, not inherited [37]. Diagnosis mainly rests on clinical features as they can determine about 95% of all cases, often eliminating the need for genetic testing. However, when needed, genetic tests can identify around 95% of NF1 mutations [37].

Anywhere from 3.9% to 25% of patients with NG1 can exhibit gastrointestinal manifestations which frequently start in the bowel with multiple present. These can be identified at an earlier age, typically around 50 years of age [5,6,38]. The most common pathologic forms of gastrointestinal involvement are ganglioneuromatosis and neurofibromatosis [39]. Intriguingly, isolated colonic neurofibromas that are not linked with NF1 or NF2 are extremely rare.

As showcased by Ghoniem et al., the presentation can vary from gastrointestinal bleeding or abdominal pain to being completely asymptomatic with the diagnosis coming as a surprise during a routine colonoscopy. When these neurofibromas are present within the colon, they are largely detected through endoscopic examinations, revealing either sessile or pedunculated lesions in the colonic mucosa or submucosa [39].

Histologically, neurofibromas manifest as proliferative entities composed of Schwann cells with characteristic wavy nuclei, axons, fibroblasts, and perineurial cells. Immunohistochemistry is essential for differentiating these tumors from other spindle-cell lesions. Neurofibromas are generally positive for S100, CD34, SOX10, and EMA [37,40].

A crucial differential diagnosis is the GIST, which can also express S100 and CD34 but will typically be positive for CD117 and almost always positive for DOG1, unlike neurofibromas [37].

While NF1-associated neurofibromas, particularly if deep-seated and multiple, carry a risk for malignant transformation/de-differentiation into malignant peripheral nerve sheath tumors (MPNSTs) [11,40], the malignancy potential for isolated colonic neurofibromas remains uncertain due to their rarity. MPNSTs are poorly differentiated, verging on undifferentiated, malignant neoplasms that are quite aggressive, sharing several characteristics with desmoplastic melanomas (including neural crest cancer stem cells as the putative histogenesis), contributing to low survival rates [40].

The clinical significance of isolated colonic neurofibromas is yet to be defined; therefore, the optimal management strategy remains uncertain. Close monitoring is advocated to both exclude the possibility of neurofibromatosis and be vigilant about the risk of malignant transformation [40].

## Conclusions

Neurofibromas, typically associated with familiar NF1 and NF2, are well-understood, benign, peripheral nerve sheath tumors. Isolated neurofibromas, especially isolated rectal neurofibromas, are extremely rare, and there have been few literature reviews in the past several decades. NF1 and NF2 are autosomal dominant genetic syndromes, which affect genetic loci 17q11.2 for NF1 and 22q 11-13.1 for NF2. Patients with NF1 and NF2 will present different clinical manifestations.

Clinical management and follow-up of isolated colonic neurofibromas remain uncertain due to their rarity and the absence of clear guidelines. Close monitoring, routine examinations, and discussions between healthcare providers and patients are crucial to understanding their clinical significance better and mitigating potential risks, including potential malignant transformation. This case contributes to our understanding of isolated colonic neurofibromas, emphasizing the need for further research and collaborative efforts to establish optimal management strategies and ensure patient well-being.
